# Caging Na_3_V_2_(PO_4_)_2_F_3_ Microcubes in Cross‐Linked Graphene Enabling Ultrafast Sodium Storage and Long‐Term Cycling

**DOI:** 10.1002/advs.201800680

**Published:** 2018-07-07

**Authors:** Yangsheng Cai, Xinxin Cao, Zhigao Luo, Guozhao Fang, Fei Liu, Jiang Zhou, Anqiang Pan, Shuquan Liang

**Affiliations:** ^1^ School of Materials Science and Engineering Central South University Changsha 410083 P. R. China; ^2^ Key Laboratory of Nonferrous Metal Materials Science and Engineering Ministry of Education Central South University Changsha 410083 Hunan China

**Keywords:** cathodes, graphene, long cycle‐life, microcubes, Na_3_V_2_(PO_4_)_2_F_3_, sodium‐ion batteries

## Abstract

Sodium‐ion batteries are widely regarded as a promising supplement for lithium‐ion battery technology. However, it still suffers from some challenges, including low energy/power density and unsatisfactory cycling stability. Here, a cross‐linked graphene‐caged Na_3_V_2_(PO_4_)_2_F_3_ microcubes (NVPF@rGO) composite via a one‐pot hydrothermal strategy followed by freeze drying and heat treatment is reported. As a cathode for a sodium‐ion half‐cell, the NVPF@rGO delivers excellent cycling stability and rate capability, as well as good low temperature adaptability. The structural evolution during the repeated Na^+^ extraction/insertion and Na ions diffusion kinetics in the NVPF@rGO electrode are investigated. Importantly, a practicable sodium‐ion full‐cell is constructed using a NVPF@rGO cathode and a N‐doped carbon anode, which delivers outstanding cycling stability (95.1% capacity retention over 400 cycles at 10 C), as well as an exceptionally high energy density (291 Wh kg^−1^ at power density of 192 W kg^−1^). Such micro‐/nanoscale design and engineering strategies, as well as deeper understanding of the ion diffusion kinetics, may also be used to explore other micro‐/nanostructure materials to boost the performance of energy storage devices.

## 1 Introduction

With the irreversible consumption of conventional fuel and the resulting environmental degradation, the energy storage and conversion technologies for sustainable and renewable energy resources are boosted rapidly.^1^ Among them, electrochemical energy storage technologies based on batteries are beginning to show considerable promise for the development of advanced power systems.^2^ Recently, SIBs with the advantages of acceptable low cost and almost analogous electrochemistry behavior to lithium‐ion batteries (LIBs) have captured attention increasingly.^3^, ^4^ However, suffered from the intrinsic defect of larger ionic radius (*R*_Na+_ = 1.02 Å vs *R*_Li+_ = 0.76 Å), there are more challenges to the cathode materials for SIBs compared to LIBs, such as unappeasable structural stability and sluggish ion diffusion resulting in undesirable sodium storage properties.^5^ To address these issues, numerous researchers are devoting themselves to discover new materials and design traditional materials to accommodate larger Na^+^ and enable highly reversible (de‐)sodiation.^5^, ^6^, ^7^

Nowadays, layered transition‐metal oxides (TMOs) and polyanion‐type compounds are two of the most typical cathode materials used for SIBs.^8^, ^9^ The TMOs, including β‐NaMnO_2_,^10^ Na*_x_*Fe*_y_*Mn*_z_*O_2_,^11^ vanadium oxides,^12^ etc., are able to deliver higher specific capacity than that of the latter, but the cycling stability of polyanion‐type compounds is much better.^6^, ^13^, ^14^, ^15^, ^16^ Intrinsically, the polyanion‐type compounds, represented by sodium superionic conductor (NASICON), possess stable 3D open framework for the rapid Na‐ion migration.^9^, ^17^ As known, Na_3_V_2_(PO_4_)_3_ (NVP) is the most typical NASICON cathode that has received extensive studies universally.[[qv: 4b,6,9,15,18]] In our previous work, the NVP nanoflakes can be charged and discharged for 30 000 cycles with a capacity retention of 62.5% at 50 C.[[qv: 4b]] Even so, the theoretical capacity of NVP is 117.6 mA h g^−1^ with an operating voltage of 3.3–3.4 V, corresponding to a theoretical energy density of about 394 W h kg^−1^.^9^ When one (PO_4_)^3−^ polyanion in NVP is substituted by three F^−^, a fluorophosphate, Na_3_V_2(_PO_4_)_2_F_3_ (NVPF), with higher theoretical energy density (about 507 Wh kg^−1^) can be obtained.^19^ The average potential and theoretical capacity of this material can achieve as high as 3.95 V and 128 mA h g^−1^, respectively.^3^, ^20^ As a member of NASICON family, NVPF also inherits the advantages of large interstitial channels and excellent structural stability.^21^ Thus, the vanadium‐based fluorophosphates with high energy densities are considered as the promising candidates for SIBs as high‐voltage cathode materials.^3^, ^17^, ^21^, ^22^, ^23^

Nevertheless, the electronic conductivity of NVPF is just 10^−12^ S cm^−1^, which greatly limits its further practical applications, especially in large‐scale energy storage systems (ESSs) when rate performance is a prerequisite.^22^, ^24^ Construction of the composite materials with carbon‐based materials is an economic and effective technique to overcome the inferior electronic conductivity, such as mesoporous carbon (CMK‐3),^22^ graphene,^3^ and other carbonaceous materials.^17^, ^25^ Among them, introduction of graphene is regarded as one of the most effective ways to elevate the electrochemical performance of electrode materials.[[qv: 4b,26]] First, it is well‐known that it can achieve enhanced electron transport capability.^27^ Second, the graphene is able to provide buffering space to accommodate the variation of stresses and volume during the ion (de)intercalation processes.^28^ The graphene wrapped structure can also inhibit material pulverization effectively.^29^ Furthermore, the graphene can also provide adequate surface areas for electrode/electrolyte contact to shorten the distance of ion diffusion.^30^

In present work, we proposed a facile hydrothermal approach with subsequent calcination to construct a novel 3D composite structure, that is, NVPF microcubes caged by interconnected reduced graphene oxides (rGO). This unique architecture can provide bicontinuous electron/ion transport pathways and large electrode–electrolyte contact area for rapid Na^+^‐ion diffusion and electron transport. Meanwhile, the 3D rGO network with robust structure stability can rapidly accommodate the volume variations during repeated Na^+^ extraction/insertion.^14^, ^31^ As cathode for both sodium‐ion half‐cell and full‐cell (NVPF@rGO||N‐doped carbon), the NVPF@rGO exhibits excellent rate performance and outstanding cycling stability.

## 2 Results and Discussion

**Figure 1:**
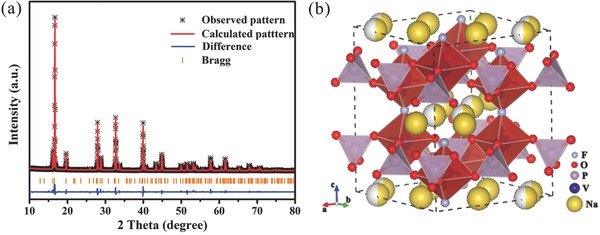
a) Rietveld refined XRD patterns of as‐prepared Na_3_V_2_(PO_4_)_2_F_3_ without carbon and b) the structural framework of Na_3_V_2_(PO_4_)_2_F_3_.

**Figure** **1**a shows the Rietveld refinement (GSAS software) of the pure NVPF on the basis of the powder X‐ray diffraction (XRD) data. All the diffraction peaks are indexed well into the tetragonal NVPF phase (*R*_p_ = 5.08%, *R*_wp_ = 8.59%) with refined parameters of space group *P4_2_*/*mnm*, *a* = *b* = 9.03 Å, *c* = 10.63 Å, and V = 868.5 Å^3^, which is in accordance with previous report.^32^ As shown in Figure 1b, the 3D framework of NVPF that contains large tunnels along the [110] and $\left[1\bar{1}0\right]$ crystal orientation is composed of V_2_O_8_F_3_ bioctahedral and PO_4_ tetrahedra. This stable structure confers NVPF open channels for rapid Na^+^ migration. In the 3D framework, there are two different positions for sodium ions which are fully occupied site and partially occupied site.^21^ The former is too stable to extract from the structure. So the electrochemical behaviors of NVPF are generally dominated by the partially occupied Na^+^.^32^

**Figure 2:**
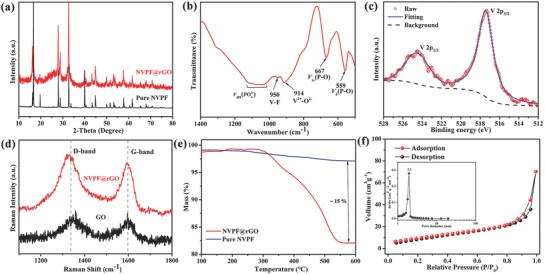
a) The XRD pattern, b) FTIR spectra, c) XPS spectrum, d) Raman scattering spectrum, e) TG curves, and f) N_2_ adsorption–desorption isotherms and corresponding BJH pore‐size distribution curve (the inset) of as‐prepared NVPF@rGO.

The XRD pattern of the NVPF@rGO composite was also collected and plotted. As shown in **Figure** **2**a, all diffraction peaks are in accord with the NVPF without carbon, indicating that there is rare impact of the rGO to the phase structure of Na_3_V_2_(PO_4_)_2_F_3_. The swell of the background around 2θ = 27° is related to the reduced GO.^33^, ^34^ The NVPF@rGO was further characterized via Fourier transform infrared (FTIR) as shown in Figure 2b. The broadband located at 1025–1125 cm^−1^ is assigned to the asymmetric stretching vibration (*vas*) P—O bonds in PO_4_ tetrahedra, while the bands at 667 and 559 cm^−1^ suggest the symmetric stretching vibration (*vss*) and bending vibration (*vb*), respectively.^9^ The band at 914 cm^−1^ can be attributed to the vibration of V_3_^+^—O_2_^−^ bonds in VO_6_ octahedra,^23^ while the band at 950 cm^−1^ demonstrates the presence of V—F bonds.^9^ The X‐ray photoelectron spectroscopy (XPS) measurements were conducted to analyze the oxidation states of elements in the as‐prepared NVPF@rGO. As shown in Figure S1 (Supporting Information), the six elemental signals (C 1s, O 1s, Na 1s, F 1s, P 2s, P 2p, and V 2p peaks) can be observed in the survey XPS spectrum of the NVPF@rGO. Couple of peaks can be observed from high‐resolution spectrum of V element (Figure 2c), one at 517 eV for V 2p_3/2_ and other at 524 eV for V 2d_1/2_, which are characteristic peaks of V^3+^ in NVPF and in good agreement with other literatures.^9^, ^35^

As shown in Figure 2d, the Raman spectrums of NVPF@rGO and pure GO are measured to investigate the state of carbon in the composite. The two broad and intense bands around 1326 and 1592 cm^−1^ can be ascribed to disordered graphitic carbon (D‐band) and sp^2^ carbon atoms (G‐band), respectively.^29^, ^34^ It can be seen that the intensity ratios of D band to G band (*I*_D_/*I*_G_) of NVPF@rGO (about 1.04) is higher than that of GO (about 1.01). The increasing *I*_D_/*I*_G_ suggests the presence of rGO.^29^, ^36^ The content of rGO in the NVPF@rGO is evaluated to be ≈15 wt% via a thermogravimetric analysis (TGA), as shown in Figure 2e. In order to characterize the specific surface area and pore structure of the composite, nitrogen adsorption–desorption technique is utilized. As presented in Figure 2f, it can be described as type‐IV isothermal adsorption–desorption curves. According to the Brunauer–Emmett–Teller (BET) method, the surface area of NVPF@rGO is 34.99 m^2^ g^−1^. The Barrett–Joyner–Halenda (BJH) pore size distribution curve (the inset) reveals that the majority pore size in NVPF@rGO is about 3.2 nm.

**Figure 3:**
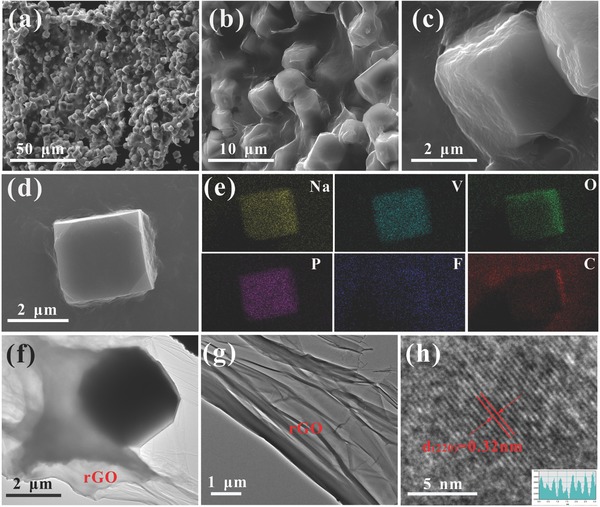
a–d) SEM images, e) elemental mapping images, f,g) TEM images, and h) HRTEM image of the as‐prepared NVPF@rGO.

The morphology and microstructure of the pure NVPF are recorded in Figure S2 (Supporting Information) via scanning electron microscopy (SEM). The as‐prepared compound presents highly uniform microcubes‐like structure with the size of 3–4 µm. When the GO was added, it can be seen that all the NVPF microcubes are wrapped by the rGO (as shown in **Figure** **3**a–c) Compared to the pure NVPF, particle size of microcubes in NVPF@rGO is reduced to ≈2 µm. It demonstrates that the introduction of rGO functions as a “confined microspace,” which effectively prevents NVPF nanocubes from growing bigger. This unique structure with 3D rGO network can modify the conductivity of NVPF and buffer the variation of stress originated from the repeated extraction/insertion of sodium ion. As present in elemental mapping images (Figure 3e), the elements of Na, V, O, P, F, and C are distributed uniformly in the profile of the composite. The contents of Na, V, P, F, and C in NVPF@rGO are 14.32, 21.16, 12.87, 11.8, and 13.17 wt%, respectively. The results not only confirm the uniform distribution of the constituting elements, but also illustrate the good quality of the composite. The transmission electron microscopy (TEM) image (Figure 3f) further indicates that the NVPF microcubes are encapsulated in rGO evenly. As shown in Figure 3g, the as‐prepared rGO is an ultrathin film with wrinkle which identifies with previous reports.^37^ In the high‐resolution TEM (HRTEM) image (Figure 3h), there are clear lattice fringes with the spacing of 0.32 nm, which can be indexed to the interplanar distance of (220) plane in tetragonal NVPF crystal.

**Figure 4:**
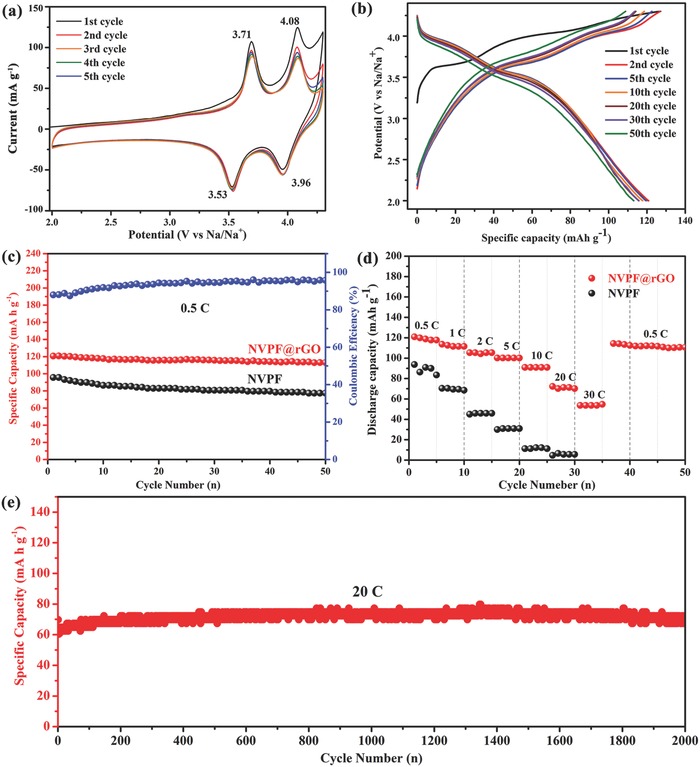
Electrochemical performance of NVPF@rGO cathode in sodium‐ion half‐cell. a) The first five successive CV curves at a scan rate of 0.1 mV s^−1^ between 2 and 4.3 V; b) the charge/discharge profiles of the selected cycles at 0.5 C; c) the cycling performance at 0.5 C; d) the rate capacity from 0.5 to 30 C; e) the long cycling performance at 20 C.

To evaluate the electrochemical properties of NVPF@rGO, a series of half cells (NVPF@rGO||Na) have been tested. **Figure** **4**a presents the initial five successive cyclic voltammetry (CV) curves, there are two pairs of cathodic peaks (located at 3.53 and 3.96 V) and anodic peaks (near 4.08 and 3.71 V), corresponding to redox reaction of V^3+^/V^4+^ with extraction/insertion of two sodium ions. The overlap of these redox peaks indicates the good reversibility of the successive cycling processes (Na_3_V_2_(PO_4_)_2_F_3_ ↔ NaV_2_(PO_4_)_2_F_3_ + 2Na^+^ + 2e^−^).^21^, ^38^ Moreover, as shown in Figure 4b, the charge/discharge profiles are basically identical for 50 cycles, suggesting the stable charge/discharge behavior.

As compared in Figure 4c, the NVPF@rGO demonstrates higher discharge capacity than that of pure NVPF, with a stable discharge capacity of 113 mA h g^−1^ for 50 cycles. The rate performances of NVPF@rGO and NVPF were also measured at consecutive current densities varied from 0.5 to 30 C (Figure 4d). The NVPF@rGO electrode delivers an average discharge capacity of 119, 111, 104, 100, 90, 71, and 53 mA h g^−1^ at corresponding rates of 0.5, 1, 2, 5, 10, 20, and 30 C, respectively. When the current density reset to 0.5 C, the specific capacity of NVPF@rGO turns back to 114 mA h g^−1^. However, the rate property of NVPF without rGO is much inferior, indicating that the introduction of rGO is beneficial to the sodium storage capability of NVPF. Specially, as shown in Figure 4e, the NVPF@rGO shows excellent long‐term cycling stability at high rate of 20 C, with stable capacity of 69 mA h g^−1^ even after 2000 cycles, corresponding to a high capacity retention of 98%. Compared to other reported Na_3_V_2_(PO_4_)_2_F_3_ cathode in sodium‐ion batteries, NVPF@rGO reported in our work demonstrates improved electrochemical performance. For example, the initial capacity of Na_3_V_2_(PO_4_)_2_F_3_ particles prepared by Song et al. is just 111.5 mA h g^−1^ at current density of 11.7 mA g^−1^.^23^ The carbon‐coated Na_3_V_2_(PO_4_)_2_F_3_ nanoparticles reported by Liu et al. exhibited excellent rate performance, but the capacity retentions at 10 and 30C are just 70 and 50%, respectively.^17^ Qi et al. have tried to improve the electrochemical performance of Na_3_V_2_(PO_4_)_2_F_3_ by fabricating nanoflowers structure, but it can only deliver a low capacity of 62 mA h g^−1^ when the rate current is increased to 5 C.^2^ Importantly, the cell can cycle at low temperature. Figure S3a (Supporting Information) presents the selected charge–discharge profiles of the first, second, and fifth cycle for the NVPF@rGO at current rate of 1 C and the testing temperature of 0 °C. There are also two couples of charge and discharge plateaus. The NVPF@rGO exhibits the initial discharge capacity of 68 mA h g^−1^ with a coulombic efficiency of 62%. After 180 cycles (Figure S3b, Supporting Information), it still maintained a considerable discharge capacity of 75 mA h g^−1^, indicating the excellent ionic diffusion kinetics and electronic conductivity of the NVPF@rGO composite.

**Figure 5:**
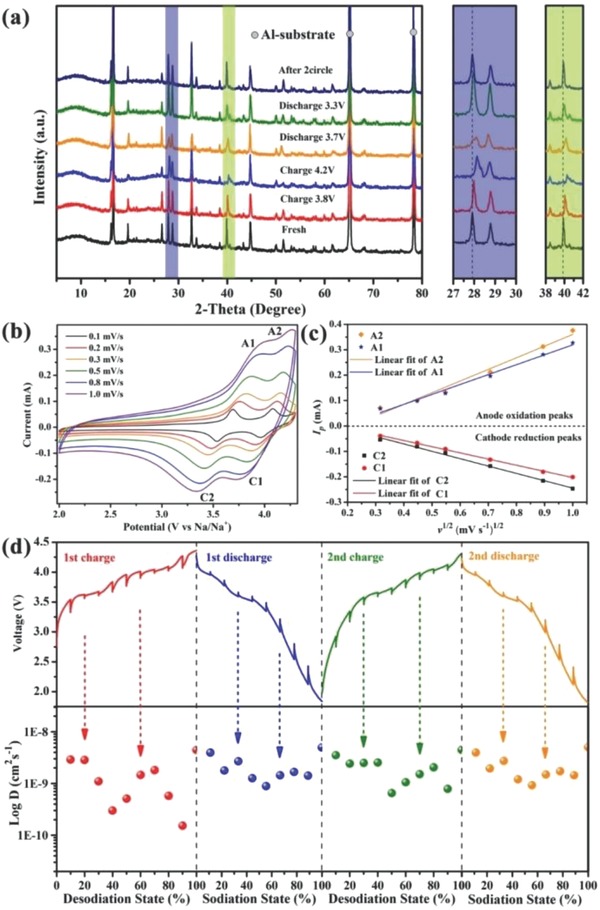
a) Ex situ XRD patterns of NVPF@rGO electrodes charged or discharged to different voltage states and after two cycles under the applied current rate of 0.5 C; b) CV curves of NVPF@rGO electrode at various scan rates; c) the line relationship between the peak current (*I*_p_) and square root of scan rate (ν^1/2^); d) GITT electrochemical charge/discharge curves of NVPF@rGO.

In order to reveal the charged/discharged mechanism of NVPF@rGO electrodes, ex situ XRD method was utilized at various voltage states. As recorded in **Figure** **5**a, all these patterns present almost coincident characteristic peaks, manifesting that the electrochemical reaction of NVPF@rGO electrodes belongs to a highly reversible (de)intercalation‐type. A couple of peaks at 2θ = 65.28° and 78.14° can be indexed to Al phase (JCPDS No. 04‐0787), which is attributed to the current collector.^39^ As presented in the highlighted images, the diffraction peaks of (220) and (040) crystal faces are located at 27.87° and 39.82° in the pattern of fresh electrode, respectively. When the NVPF@rGO electrodes are charged to 3.8 V, both of the two peaks shift to higher angles, corresponding to the two‐phase transformation from Na_3_V_2_(PO_4_)_2_F_3_ to Na_2_V_2_(PO_4_)_2_F_3_ with the extraction of Na ions at fully occupied positions. As the voltage achieves 4.2 V, the positions of the two peaks are changed even more, which indicates that another Na ion is extracted through the transformation from Na_2_V_2_(PO_4_)_2_F_3_ to Na_1_V_2_(PO_4_)_2_F_3_.^20^, ^38^ It can be observed that the intensities of some diffraction peaks weakened gradually during the charge process, suggesting that the structure of Na_1_V_2_(PO_4_)_2_F_3_ is less stable than that of Na_3_V_2_(PO_4_)_2_F_3_. However, the diffraction peaks can be recovered as the NVPF@rGO electrodes discharging to 3.7 V. The result demonstrates that the electrochemical behavior of NVPF@rGO electrodes is a reversible process of Na^+^ extraction/insertion (Na_3_V_2_(PO_4_)_2_F_3_ ↔ Na_1_V_2_(PO_4_)_2_F_3_ + 2Na^+^ + 2e^−^). As shown in Figure S4a (Supporting Information)**,** even after 50 cycles, the cathode still maintains the microcube‐like structure coated by rGO. The lattice fringes with a spacing of 0.202 nm in the HRTEM image (Figure S4b, Supporting Information) are related to the distance of (240) plane in NVPF crystal, suggesting the presence of Na_3_V_2_(PO_4_)_2_F_3_ phase. Thus, NVPF@rGO composite is a promising cathode with electrochemically stable crystallinity and robust structure stability for SIBs.

CV and galvanostatic intermittent titration technique (GITT) technologies are utilized to further explore the Na‐migration kinetics in the NVPF@rGO electrode (more details are described in the Supporting Information). As shown in Figure 5b, the electrodes are cycled in the voltage range of 2.0–4.3 V with scan rates of 0.1, 0.3, 0.3, 0.5, 0.8, and 1.0 mV s^−1^. When the scan rates are increased, not only the area of the CV curves but also the intensities and positions of redox peaks are changed, which is ascribed to enlarged polarization.^40^ Figure 5c presents the linear relation between the peak current (*I*_p_) and square root of scan rate (ν^1/2^) based on Figure 5b. The good fitting results demonstrate that the electrochemical behavior of NVPF@rGO electrodes is a diffusion‐controlled process. Thus, the Randles–Sevcik equation^9^ can be employed to carry out the diffusion coefficients of sodium ion (*D*). The *D* values are 5.13 × 10^−11^, 1.58 × 10^−10^, 2.52 × 10^−10^, and 4.44 × 10^−11^ cm^2^ s^−1^, corresponding to the redox peaks of A1, A2, C1, and C2, respectively. The redox couple at high potential (A2/C1) exhibits larger *D* values than that at low potential (A1/C2), indicating the better ability of sodium‐ion diffusion in the composite at a high potential (about 4.0 V).

Moreover, Figure S5 (Supporting Information) presents single titration curves of charge and discharge processes during GITT measurements with a pulse time of 600 s at 0.5 C, followed by a relaxation time of 1800 s. The slow changes of potential are related to the diffusion of sodium ion, while the sharp increase or decrease can be ascribed to charge transfer and ohm resistance.^41^ The variation of the effective diffusion coefficients (*D*_e_) for NVPF@rGO electrodes from first charge process to second discharge are shown in Figure 5d. The values of *D*_e_ are worked out in the order magnitude of 10^−9^–10^−10^ cm^2^ s^−1^, indicating a fast diffusion behavior of this NASICON‐type composite. During charge (discharge) processes, the values at low‐potential plateaus around 3.7 V (3.5 V) are evidently smaller than that of high‐potential plateaus at about 3.9 V (4.1 V), which is similar to the CV results. In addition, the *D*_e_ of NVPF@rGO electrode is higher than that of many other NASICON‐type phosphates,^9^, ^42^ suggesting the excellent ionic diffusion kinetics of the NVPF with 3D rGO capping.

**Figure 6:**
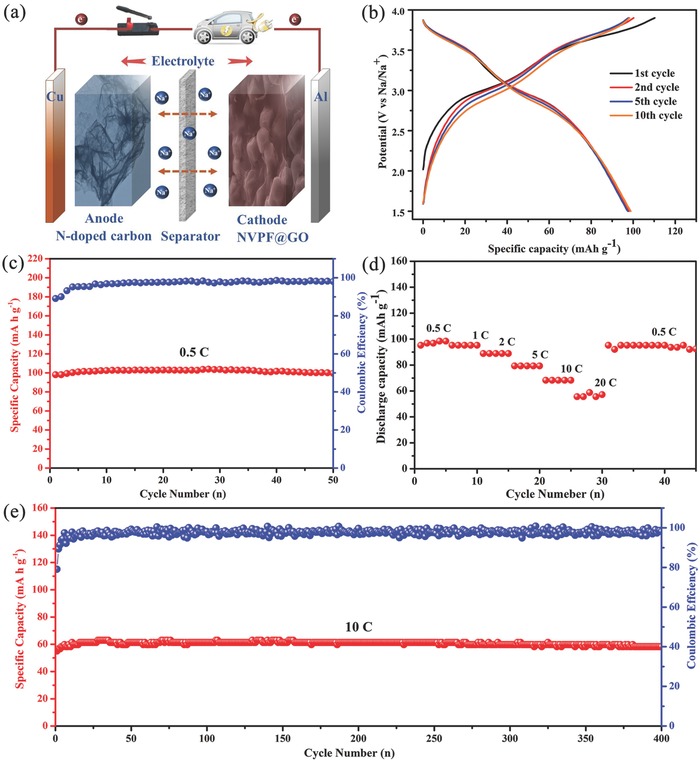
Electrochemical performance of NVPF@rGO cathode in sodium‐ion full‐cell. a) Schematic of the sodium‐ion full‐cell (NVPF@rGO||N‐doped carbon); b) the charge/discharge profiles of the selected cycles at the current density of 0.5 C; c) the cycling performance at 0.5 C; d) the rate performance from 0.5 to 20 C; e) the long cycling performance at 10 C.

To further demonstrate the practical feasibility of the prepared composite, sodium‐ion full‐cell (NVPF@rGO||N‐doped carbon) has been assembled by employing N‐doped carbon nanosheets anode reported by our group,^43^ as illustrated in **Figure** **6**a. The selected charge/discharge profiles of the full‐cell at a current rate of 0.5 C (voltage window of 1.5–3.9 V) is shown in Figure 6b. The respective charge and discharge capacities of the full‐cell at first cycle are 110 and 98 mA h g^−1^ with a coulombic efficiency of 89%. There are two obvious charge plateaus at 3.02 and 3.68 V with the corresponding discharge plateaus at 2.96 and 3.57 V. From the second to the tenth cycle, the charge/discharge curves are almost identical, demonstrating a highly reversible charge/discharge behavior. After 50 cycles, it maintains a discharge capacity as high as 99.6 mA h g^−1^ (Figure 6c). The rate capability of the full‐cell has also been evaluated at consecutive current rates varied from 0.5 to 20 C (Figure 6d), with an average discharge capacity of 96, 95, 88, 79, 68, and 55 mA h g^−1^ at corresponding rates of 0.5, 1, 2, 5, 10, and 20 C, respectively. When the current rate turns back to 0.5 C, the specific capacity of the full‐cell is 95 mA h g^−1^. The excellent rate performance can be attributed to the good ability of sodium‐ion diffusion in the electrode materials. Moreover, the long‐term cycling measurement of the sodium‐ion full‐cell at current rate of 10 C is presented in Figure 6e. Before 15 cycles, the discharge capacity is gradually increased from 55 to 61 mA h g^−1^. It can hold a capacity of 58 mA h g^−1^ even after 400 cycles, which further confirms the outstanding stability of the full‐cell. Figure S6 (Supporting Information) shows the Ragone plot of the present work and other phosphate‐based full‐cells (normalized to the weight of cathode materials). This NVPF@rGO||N‐doped carbon full‐cell possesses a high energy density of 291 W h kg^−1^ at a low power density of 192 W kg^−1^. When the power density is increased to 6144 W kg^−1^, the energy density can still maintain 139 W h kg^−1^. In comparison, although the energy density of this cell is lower than that of VOPO_4_/Na_2_Ti_3_O_7_ at power density of <100 W kg^−1^,^44^ the present device obviously exhibits more desirable power performance,^15^, ^16^, ^44^, ^45^ suggesting the NVPF@rGO is highly desirable for high‐power SIBs applications such as hybrid electric vehicles, electric vehicles, and large‐scale ESSs. On the basis of above results, the NVPF@rGO with outstanding electrochemical performance is a promising cathode material for SIBs, owing to the upgrades of electron conductivity, fast ion diffusion capability, and structural stability by cross‐linked 3D graphene wrapped NVPF.

## 3 Conclusion

In summary, NVPF@rGO microcubes encapsulated in 3D graphene network have been successfully prepared via a one‐pot hydrothermal strategy with subsequent freeze drying and heat treatment. As a cathode for sodium‐ion half‐cell, the NVPF@rGO cathode exhibits enhanced cycling stability and improved rate performance. Even tested in 0 °C, it delivers 75 mA h g^−1^ even after 180 cycles at a rate of 1C. The ex situ XRD and TEM techniques disclose the excellent reversibility and stability of the composites during the process of Na^+^ extraction/insertion. CV and GITT technologies are utilized to illustrate the favorable Na^+^ diffusion kinetics in the NVPF@rGO electrode. Importantly, the sodium‐ion full‐cell with NVPF@rGO as cathode and N‐doped carbon as anode demonstrates highly reversible sodium storage capability and desirable power performance. These encouraging results may accelerate further development of SIBs by micro‐/nanoscale design and engineering of the electrode materials.

## 4 Experimental Section

**Scheme 1:**
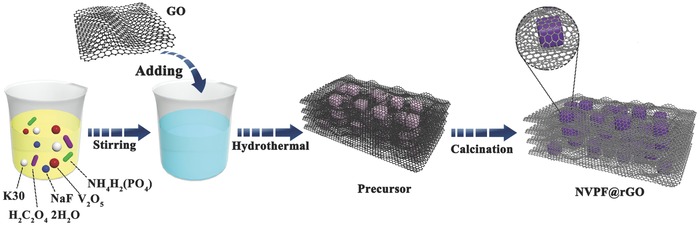
Preparation procedure of Na_3_V_2_(PO_4_)_2_F_3_ microcubes/graphene composites.

*Materials Synthesis*: The preparation procedure of Na_3_V_2_(PO_4_)_2_F_3_ microcubes wrapped by rGO is illustrated in **Scheme** **1**.

All the raw materials, including V_2_O_5_, H_2_C_2_O_4_·2H_2_O, NH_4_H_2_PO_4_, and NaF, are analytical grade. Typically, 182 mg V_2_O_5_ and 324 mg H_2_C_2_O_4_ 2H_2_O were dissolved in 15 mL deionized water under vehemently stirring at 70 °C for 20 min. Successively, 230 mg NH_4_H_2_PO_4_ and 126 mg NaF were added into the blue solution under stirring successively. After another 20 min, 500 mg polyvinyl pyrrolidone (K30), which is used to construct gel‐like 3D graphene, was added to the above solution under stirring until it dissolved completely. Then, 15 mL graphene oxide (GO) suspension (≈2 mg mL^−1^), which was obtained by a modified Hummers' method,^46^ was poured into the prepared solution with stirring for 15 min and sonication for 10 min. The black mixture was transferred to a 50 mL Teflon‐lined stainless steel autoclave and kept in an electrical oven at 170 °C for 9 h. When it cooled down to room temperature naturally, the obtained gel was dried by a freeze dryer. According to the thermogravimetric analyses (Figure S7, Supporting Information), the precursor was annealed in Ar atmosphere at 480 °C for 8 h to get the final product. Pure NVPF was also prepared through the same solvothermal conditions, freeze‐drying, and calcination process as applied in the synthesis of NVPF@rGO except without adding polyvinyl pyrrolidone and GO.

*Material Characterization*: The crystallographic phases of the as‐prepared powder and electrodes were determined by a Rigaku D/max 2500 X‐ray diffractometer with Cu Kα radiation. The morphology and structural characterization of the composites and cycled electrodes were recorded through SEM (FEI Nova Nano‐SEM 230) and TEM (JEOL‐JEM‐2100F). FTIR spectroscopy (AVTATAR, 370) was employed to probe the chemical bonds in the NVPF@rGO. XPS measurements were performed to probe the oxidation states of elements in the surface. The structure and content of carbon were measured by a Raman spectroscopy (LabRAM Hr800) and TGA (Q500), respectively. The BET surface area of the composite was analyzed by nitrogen adsorption–desorption isotherms, which was obtained from a NOVA 4200e surface area and pore size analyzer (Quantachrome Instruments). A combined C‐S analyzer and an ICP test were performed to investigate the content of elements in NVPF@rGO.

*Electrode Fabrication and Electrochemical Measurement*: In order to evaluate the electrochemical performance of the composite, CR 2016 coin cells were assembled in the Mbraun glove box (Made in Germany). A piece of aluminum foil, which was coated viscous slurry (active materials:acetylene black:polyvinylidene fluoride binder = 7:2:1, in weight), was used as cathode. Sodium metal plates and glass fiber were employed as anode and separator, respectively. A commercial electrolyte, containing 1 m NaClO_4_ dissolved in ethylene carbonate/dimethyl carbonate (EC:DMC = 1:1, in volume) with 5% fluoroethylene carbonate (FEC), was selected. The capacity and current density are based on the mass of NVPF materials only. The areal loading of cathode materials for each electrode in this work is 0.9–1.2 mg cm^−2^. The CV and electrochemical impedance spectrometry (EIS) of all cells were measured using a multichannel electrochemical station (Multi Autolab/M 204, Metrohm). Multichannel battery testing system (Land CT 2001A, China) was utilized to analyze the galvanostatic charge/discharge behavior of the cells.

## Conflict of Interest

The authors declare no conflict of interest.

## Supporting information

Supplementary
