# Supplementary material for: Caging Na3V2(PO4)2F3 Microcubes in Cross‐Linked Graphene Enabling Ultrafast Sodium Storage and Long‐Term Cycling
Source: Adv Sci (Weinh). 2018 Jul 7;5(9):1800680. doi: 10.1002/advs.201800680 (PMC6145241; doi:10.1002/advs.201800680)
Supplement: Supplementary file 1 — Supplementary [file ADVS-5-1800680-s001.pdf]

Copyright WILEY-VCH Verlag GmbH & Co. KGaA, 69469 Weinheim, Germany, 2018.

Supporting Information

for *Adv. Sci.,* DOI: 10.1002/advs.201800680

Caging $Na_3V_2(PO_4)_2F_3$ Microcubes in Cross-Linked Graphene Enabling Ultrafast Sodium Storage and Long-Term Cycling

Yangsheng Cai, Xinxin Cao, Zhigao Luo, Guozhao Fang, Fei Liu, Jiang Zhou, Anqiang Pan, and Shuquan Liang**

DOI: 10.1002/ advs.201800680
Article type: Full Paper

Copyright WILEY-VCH Verlag GmbH & Co. KGaA, 69469 Weinheim, Germany, 2013.

Supporting Information for

Caging $Na_3V_2(PO_4)_2F_3$ microcubes in cross-linked graphene enabling ultra-fast sodium storage and long-term cycling

*By Yangsheng Cai, Xinxin Cao, Zhigao Luo, Guozhao Fang, Fei Liu, Jiang Zhou**, *Anqiang Pan, and Shuquan Liang**

Y. Cai, X. Cao, Z. Luo, G. Fang, F. Liu, Prof. J. Zhou, Prof. A. Pan, and Prof. S. Liang
School of Materials Science and Engineering
Central South University, Changsha 410083, P. R. China

Prof. J. Zhou, Prof. A. Pan, and Prof. S. Liang
Key Laboratory of Nonferrous Metal Materials Science and Engineering, Ministry of Education, Central South University, Changsha 410083, Hunan, China

E-mail address: zhou_jiang@csu.edu.cn, lsq@csu.edu.cn

Keywords: $Na_3V_2(PO_4)_2F_3$, Microcubes, Graphene, Cathode, Long-cycle-life, Sodium-ion batteries

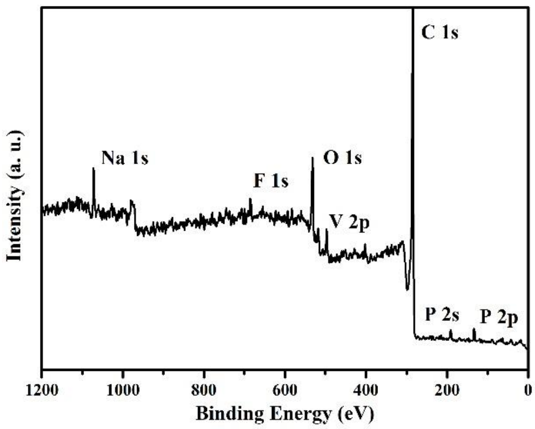

Figure S1. The survey XPS spectrum of the NVPF@rGO.

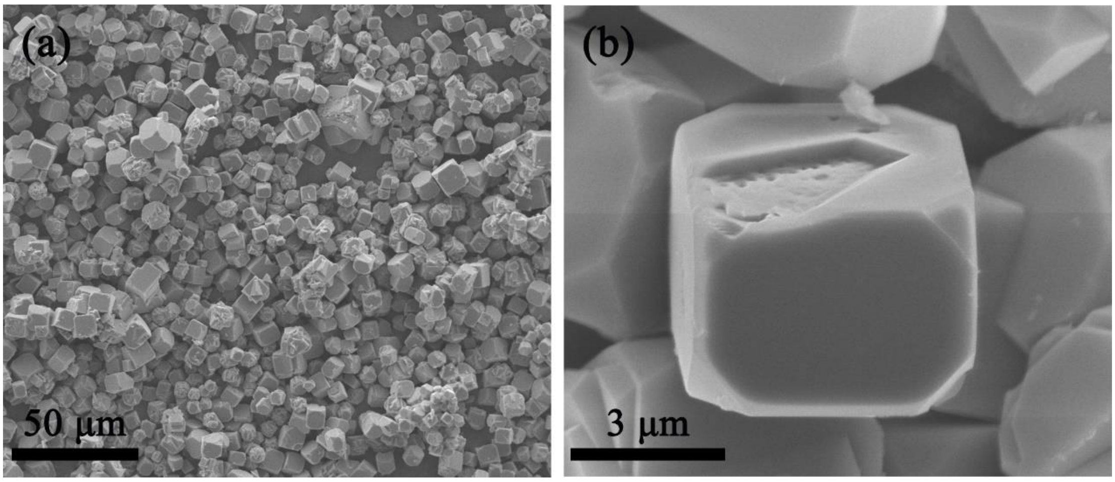

Figure S2. (a, b)The SEM images of pure $Na_3V_2(PO_4)_2F_3$.

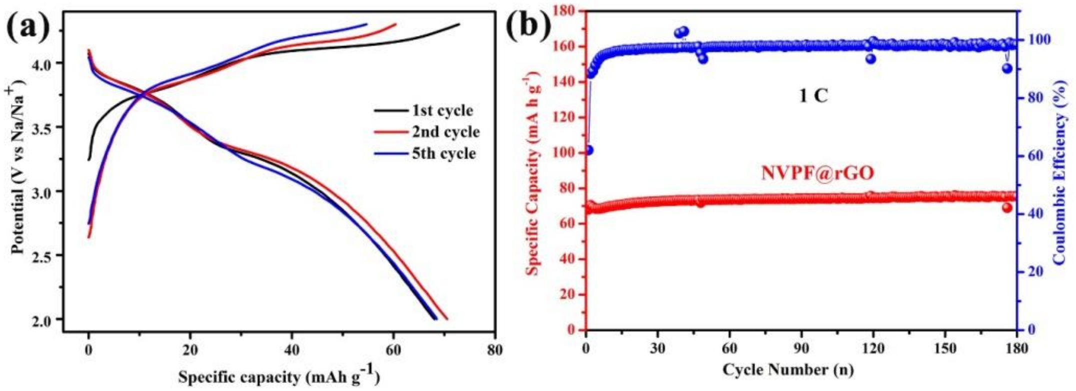

Figure S3. (a) The charge/discharge profiles of the selected cycles and (b) cycling performance of NVPF@rGO at 1 C and the testing temperature of 0 ℃.

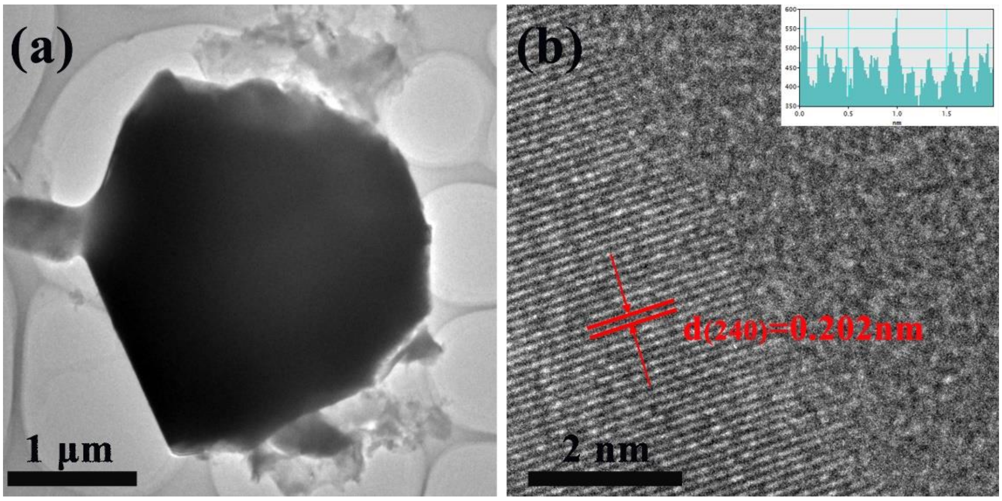

Figure S4. (a) TEM image and (b) HRTEM image of NVPF@rGO after 50 charge-discharge cycles at current rate of 0.5 C.

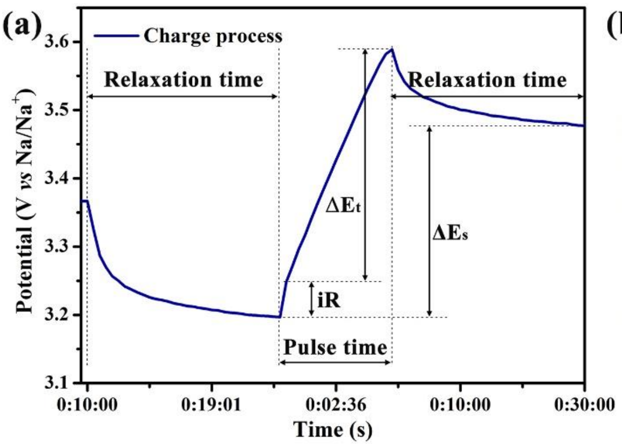

Figure S5. Potential *vs.* time curves of the NVPF@rGO electrode for a single GITT during (a) charge and (b) discharge processes.

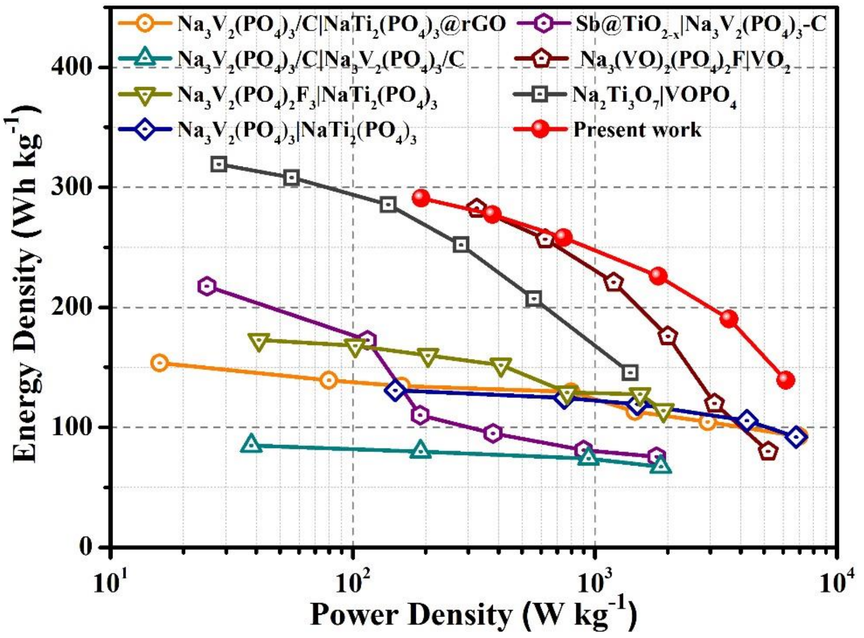

Figure S6. The Ragone plots of present work and other phosphate-based full-cells.[1-7]

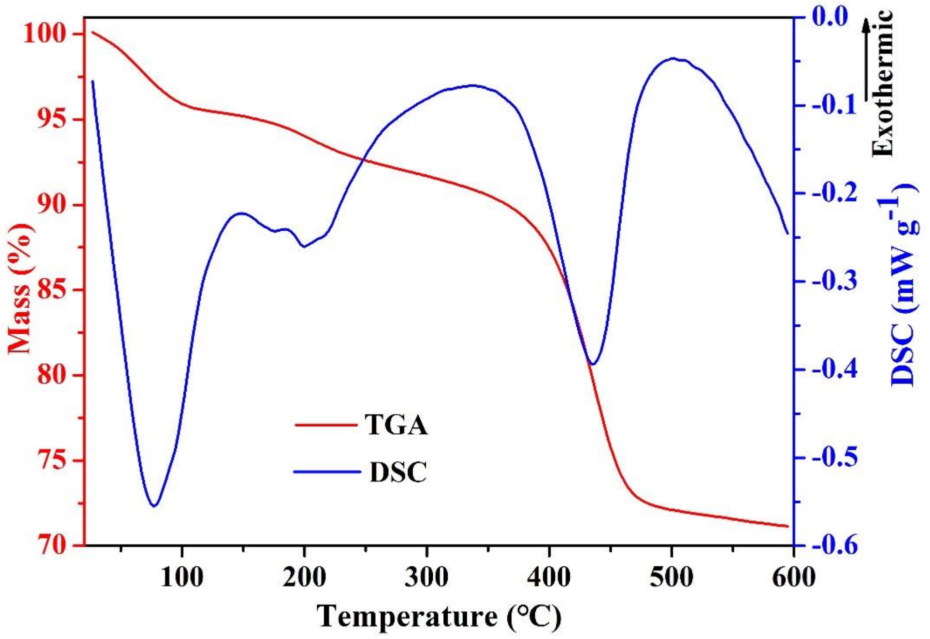

Figure S7. The thermogravimetric analyses of the precursor.

Reference:

1. Li, H.; Peng, L.; Zhu, Y.; Chen, D.; Zhang, X.; Yu, G. An Advanced High-Energy Sodium Ion Full Battery Based on Nanostructured $Na_2Ti_3O_7/VOPO_4$ Layered Materials. *Energ. Environ. Sci.* **2016**, *9*, 3399-3405.
2. Wang, N.; Bai, Z.; Qian, Y.; Yang, J. Double-Walled Sb@TiO_{2-x} Nanotubes as a Superior High-Rate and Ultralong-Lifespan Anode Material for Na-Ion and Li-Ion Batteries. *Adv. Mater.* **2016**, *28*, 4126-4133.
3. Chao, D.; Lai, C.-H. M.; Liang, P.; Wei, Q.; Wang, Y.-S.; Zhu, C. R.; Deng, G.; Doan-Nguyen, V. V. T.; Lin, J.; Mai, L.; Fan, H. J.; Dunn, B.; Shen, Z. X. Sodium Vanadium Fluorophosphates (NVOPF) Array Cathode Designed for High-Rate Full Sodium Ion Storage Device. *Adv. Energy Mater.* **2018**, 1800058. (https://doi.org/10.1002/aenm.201800058)
4. Saravanan, K.; Mason, C. W.; Rudola, A.; Wong, K. H.; Balaya, P. The First Report on Excellent Cycling Stability and Superior Rate Capability of $Na_3V_2(PO_4)_3$ for Sodium Ion Batteries. *Adv. Energy Mater.* **2013**, *3*, 444-450.
5. Fang, Y.; Xiao, L.; Qian, J.; Cao, Y.; Ai, X.; Huang, Y.; Yang, H. 3D Graphene Decorated $NaTi_2(PO_4)_3$ Microspheres as a Superior High-Rate and Ultracycle-Stable Anode Material for Sodium Ion Batteries. *Adv. Energy Mater.* **2016**, *6*, 1502197.
6. Chihara, K.; Kitajou, A.; Gocheva, I. D.; Okada, S.; Yamaki, J.-i. Cathode Properties of $Na_3M_2(PO_4)_2F_3$ [M=Ti, Fe, V] for Sodium-Ion Batteries. *J. Power Sources* **2013**, *227*, 80-85.
7. Ren, W.; Zheng, Z.; Xu, C.; Niu, C.; Wei, Q.; An, Q.; Zhao, K.; Yan, M.; Qin, M.; Mai, L. Self-Sacrificed Synthesis of Three-Dimensional $Na_3V_2(PO_4)_3$ Nanofiber Network for High-Rate Sodium–Ion Full Batteries. *Nano Energy* **2016**, *25*, 145-153.
